# Synthetic fibrosis distributions for data augmentation in predicting atrial fibrillation ablation outcomes: an *in silico* study

**DOI:** 10.3389/fcvm.2025.1512356

**Published:** 2025-04-11

**Authors:** Alexander M. Zolotarev, Kiane Johnson, Yusuf Mohammad, Omnia Alwazzan, Gregory Slabaugh, Caroline H. Roney

**Affiliations:** ^1^ School of Engineering and Materials Science, Queen Mary University of London, London, United Kingdom; ^2^ Queen Mary’s Digital Environment Research Institute (DERI), London, United Kingdom; ^3^ School of Electronic Engineering and Computer Science, Queen Mary University of London, London, United Kingdom

**Keywords:** atrial fibrillation, ablation, diffusion models, multi-modal fusion, computer vision, biophysical simulations

## Abstract

**Introduction:**

Cardiac fibrosis influences atrial fibrillation (AF) progression and ablation outcomes, with late gadolinium enhancement (LGE) MRI providing a non-invasive tool to measure fibrosis distributions. While deep learning (DL) has shown promise in predicting ablation success, training such pipelines is limited by the availability of real patient data.

**Methods:**

In this study, we generated synthetic fibrosis distributions using a denoising diffusion probabilistic model trained on a collection of 100 real LGE-MRI distributions. We incorporated them into 1,000 bi-atrial meshes derived from a statistical shape model and simulated AF episodes on them before and after various ablation strategies to expand the training dataset for DL-based outcome prediction. Our approach aims to improve the predictive performance of the DL pipeline by enhancing dataset diversity and better-capturing patient variability.

**Results:**

We showed that the fibrosis distributions generated by the diffusion model closely resemble real LGE-MRI distributions, based on metrics such as mean intensities (1.1±0.2 vs. 1.1±0.3) and average Shannon entropy (0.77±0.06 and 0.81±0.03). AF biophysical simulations can be effectively conducted on bi-atrial meshes incorporating these synthetic distributions. Training the deep learning pipeline on these simulations produces performance metrics comparable to those achieved with real LGE-MRI distributions (ROC-AUC = 0.952 vs. 0.943).

**Conclusion:**

We have shown the ability of synthetic fibrosis distributions to be a data augmentation tool for deep learning classification of outcomes of various ablation strategies, which may enable rapid and precise assessment of atrial fibrillation treatment strategies.

## 1 Introduction

Cardiac fibrosis distributions vary uniquely between patients, influencing patterns of electrical activity (1, 2). Late gadolinium enhancement (LGE) MRI offers a non-invasive method for quantitatively assessing fibrosis. Gadolinium accumulates in fibrotic tissue more than in healthy tissue or the blood pool, leading to higher intensity in scarred areas of LGE-MRI images. The image intensity ratio (IIR), which is the ratio of MRI intensity to that of the blood pool, allows for standardized quantification of atrial fibrosis across patients and MRI systems (3). Fibrosis distributions are then used to adjust the ionic and conductivity properties of fibrotic regions, simulating atrial remodeling.

Diffusion models are successful in the generation of artificial images in various computer vision and augmented reality applications (4, 5). In cardiac basic science, Baranwal et al. (6) utilized a denoising diffusion probabilistic model (DDPM) to generate the electrical wave patterns in 2D isotropic medium and 3D bulk in time. The generation of synthetic full spatial cine cardiac magnetic resonance images via latent denoising diffusion implicit models has been shown to produce relevant results in terms of image fidelity and realism of cardiac volumes (7). Among other generative techniques, variational autoencoders (VAEs) have recently been proposed for generating realistic atrial representations that can capture patient variability in atrial anatomy. Beetz et al. (8) used VAEs to artificially generate 3D surface meshes of the ventricles as a virtual patient cohort, and Dou et al. (9) used the same type of model with dependent and independent generators to combine anatomical structures into one mesh. Finally, models proposed by Kong et al. (10) and Qiao et al. (11) can capture variability in cardiac shape for highly varied hearts with congenital diseases or construct artificial cardiac shapes with given clinical conditions.

In addition, there are several examples of successful implementation of different statistical and deep learning techniques for generating fibrosis distributions. Clayton (12) used a Gaussian random field with different length scales and showed the dependence between these lengths and the vulnerability of re-entrant activity. To address the lack of histological images with fibrosis, Lawson et al. (13) implemented a Perlin noise generator for the creation of synthetic distributions and demonstrated the effective capturing of fibrosis textures. Finally, our recent proof-of-concept abstract Zolotarev and Roney (14) used above mentioned DDPM to create realistic representations of LGE-MRI fibrosis distributions.

Fibrosis distributions influence the electrical activity through cardiac tissue. They can form conduction abnormalities for re-entry loops in atrial fibrillation (AF) (15), which remains the most common heart rhythm disorder and is associated with an increased risk of stroke (16). The surgical treatment of AF is ablation, which aims to isolate the pathological sources of AF signals. The recommended baseline strategy, as outlined in clinical guidelines, is pulmonary vein isolation (PVI). This involves using radiofrequency catheter ablation to electrically isolate the pulmonary veins (PVs) from the heart, preventing AF triggers from the PVs from propagating to the left atrial body (17). However, PVI alone is often insufficient, and the long-term success rate for persistent AF ablation therapy is generally between 50% and 60% (16, 18). It is challenging to personalize therapy to a patient because of the large variability between patients.

One possible solution for this challenge is to use cardiac digital twins and AF biophysical simulations to develop and test the efficacy of personalized ablation treatment approaches. Personalized biophysical models are based on differential equations for signal wavefront propagation coupled to a human atrial cell model and solved on patient-specific atrial anatomies (meshes constructed from imaging data). AF biophysical simulations can be run before and after possible ablation strategies to test whether AF terminates after different ablation approaches.

However, running biophysical simulations is a time-consuming process, and these cannot be performed on clinical timescales. This limitation can be overcome by using deep learning (DL) algorithms. The DL pipeline learns the patterns for successful prediction of ablation outcome during the training stage of pipeline development and then predicts the outcome for a new patient based on the learnt knowledge and the patient-specific feature maps. The ground truth (GT) ablation outcomes for DL training are obtained from AF biophysical simulations after simulating ablation approaches. The pipeline predicts the probability of AF termination based on feature maps of anatomical (for example, fibrosis distribution) and physiological (frequency and phase maps) modalities and on the ablation mask itself. However, it is challenging to collect enough data to train deep learning pipelines using clinical data alone. With this motivation, we aim to evaluate whether adding artificially generated cases improves the performance of the pipeline.

In the current study, we aimed to generate synthetic atrial fibrosis distributions via diffusion models to increase our training dataset size by imitating independent personalized AF episodes. Specifically, a DDPM (19) was trained on real LGE-MRI distributions to generate synthetic ones using a 2D representation of the atrium [*universal atrial coordinates* (UACs) (20)]. AF episodes before and after PVI and other ablation strategies were simulated on bi-atrial 3D surface atrial meshes incorporating these fibrosis maps.

We analyzed the generated distributions by applying a DL multi-class classifier to predict the outcomes for various ablation strategies. Fibrosis and other feature maps extracted from pre-ablation AF simulations were used as inputs to predict the ablation outcome. Overall, we hypothesize that synthetic fibrosis distributions correspond well with the real LGE-MRI ones and can be used for dataset expansion to improve the predictive metrics.

## 2 Methods

We have separated the Methods section into two main parts: the first will describe the process and analysis of artificially generated fibrosis distributions, and the second will cover the dataset, biophysical simulations, and deep learning pipeline.

### 2.1 Artificially generated fibrosis distributions

#### 2.1.1 Diffusion models

The core mechanism of diffusion models is the generation of synthetic images by restoring them from noise distributions. Gaussian noise is gradually added to the training images, and the model learns to reverse this process (19). Specifically, DDPMs are generative models that incrementally add noise to data in a controlled manner and then learn to reverse this process to generate new samples. The training of DDPMs involves two main steps: the forward (noising) and the reverse (denoising) processes. In the forward process, Gaussian noise is gradually added to a data sample over T time steps (Equation 1). For a data sample ***bf*** $\;x_{0}$, the forward process generates noisy samples $x_{1},x_{2},\ldots ,x_{T}$, where each noisy sample $x_{t}$ is generated by adding noise to the previous sample $x_{t-1}$:

(1) $$q(x_{t}|x_{t-1})=N(x_{t};\sqrt{1-\beta_{t}}\;x_{t-1},\beta_{t}\;I),$$

where $\beta_{t}$ is the variance scheduler that controls the amount of noise added at each step. Over multiple steps, the sample becomes entirely random noise.

The reverse process aims to recover the data by sequential denoising from $x_{T}$ to $x_{0}$ Equation 2. This is achieved by parameterizing a neural network to approximate the reverse conditional probabilities:

(2) $$p_{\theta }(x_{t-1}|x_{t})=N(x_{t-1};\mu_{\theta }(x_{t},t),\sigma_{t}^{2}\;I),$$

where $\mu_{\theta }$ is the predicted mean of the denoised sample and $\sigma_{t}^{2}$ is the variance. By learning this reverse process, DDPMs can efficiently generate realistic data samples from random noise.

For the generation of synthetic fibrosis distributions, we used the DDPM implementation from MONAI Generative Models software (21). The training was carried out on the NVIDIA GeForce RTX 3080 video card for 500 epochs using the mean squared error loss and Adam optimizer with a learning rate of 2.5 × 10^−5^. As a back bone architecture, we used the default model, i.e., the original DDPM scheduler containing 1,000 timesteps in its Markov chain, and a 2D U-Net with attention mechanisms in the second and third levels, each with one attention head.

**Figure 1:**
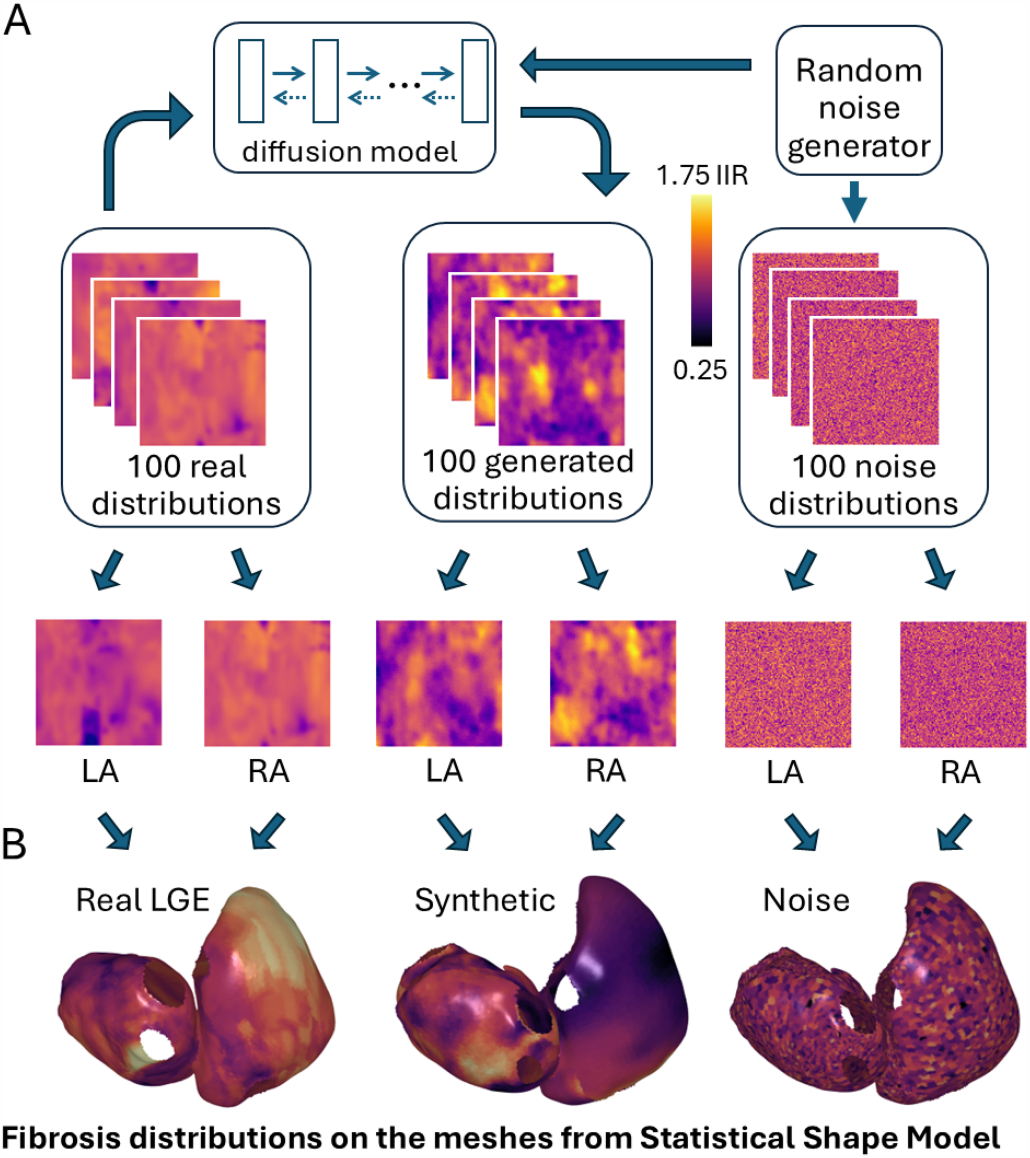
**(A)** A diffusion model was trained on real LGE-MRI distributions and generated synthetic fibrosis distributions from Gaussian noise. **(B)** These fibrosis distributions were incorporated into bi-atrial meshes derived from a statistical shape model. LA/RA, left/right atrium.

The diffusion model was trained on 100 original LGE-MRI distributions in 2D in the format of 96-by-96-pixel maps (Figure 1A). They were obtained from the collection of 100 clinical LGE-MRI scans of 100 patients with AF (43 paroxysmal and 57 persistent) undergoing first-time ablation (22). Cardiac magnetic resonance imaging was performed on 1.5 T Ingenia (Philips Healthcare, Best) and Aera Magnetom (Siemens, Erlangen) scanners. LGE imaging was performed 20 min after contrast administration using an ECG-triggered, respiratory navigated gradient echo sequence (spatial resolution 1.3 mm^3^ × 1.3 mm^3^ × 4 mm^3^ reconstructed to 1.3 mm^3^ × 1.3 mm^3^ × 2 mm^3^). The left atrium was segmented from a contrast-enhanced MRA and then registered with the corresponding LGE-MRI scan using CEMRGApp (23).

#### 2.1.2 Noise fibrosis distributions

To compare the generated cases statistically with some baseline cases with random distributions, we also generated a collection of 100 noise distributions sampled from one Gaussian distribution by using the torch.randn_like command from PyTorch library (24). The intensities were normalized between 0.25 and 1.75 IIR to match the same range of intensities for synthetic distributions.

#### 2.1.3 Statistical assessment

We used Shannon entropy (SE, Equations 3 and 4) to statistically assess the image complexity and for comparison of the synthetic cases with real LGE-MRI ones, as proposed in Lawson et al. (13). This metric is translation invariant, allowing images with similar patterns but in different areas to be assessed accurately.

(3) $$SE=\frac{-1}{\log_{2}\;(N_{x}N_{y})}\sum_{i=1}^{N_{x}}\sum_{\;j=1}^{N_{y}}P_{ij}\log_{2}\;P_{ij},$$

(4) $$P_{ij}=\frac{S_{ij}}{\sum_{i=1}^{N_{x}}\sum_{\;j=1}^{N_{y}}S_{ij}}.$$

SE is a measure of the amount of information stored in data (25). From Equation 3, $N_{x},N_{y}$ are the number of pixels in the image and are used as normalizing factors, bounding the SE values to [0,1]. A low SE means there is little information or uncertainty in the image and a high SE means there is a lot of information or uncertainty present. We calculated the SE values after applying a Gaussian filter with kernel 3 to the image. Image noise will be uniform after it, with almost the same intensity for all pixels, and without recognizable clusters, SE should be less for such an image. In contrast, images with meaningful content will have several clusters of different colors. In our case, we wanted the SE of synthetic cases to match the SE values of real LGE-MRI cases. To achieve this we excluded any synthetic case with an SE ≤0.66. The filtering helps remove low-quality cases. By ensuring a close match in SE value across real and generated cases, we can conclude our generated samples match the properties of real samples. Shannon entropy and IIR values are presented as mean± SD.

### 2.2 Atrial fibrillation predictions

#### 2.2.1 Dataset

We used 1,000 bi-atrial meshes from Roney et al. (26), which were obtained from a statistical shape model derived from cardiac computed tomography (CT) scans of 19 healthy patients (27). Anatomical structures such as the sinoatrial node, pectinate muscles, and fiber fields were added to the bilayer meshes from a bilayer atlas mesh using *UACs* (26).

We then assigned each mesh with two randomly selected fibrosis distributions for the left and right atria (LA/RA, Figure 1B). The virtual cohort was separated into four parts: a baseline training set (mesh indexes ∈ [1, 400]), a comparison training set (mesh indexes ∈ [401, 800]), a validation set (indexes ∈ [801, 900]), and a testing set (indexes ∈ [901, 1,000]). The first training set, validation, and testing sets were covered with real LGE-MRI distributions. The assignment of fibrotic remodeling properties to atrial meshes was affected by the resolution of the LGE-MRI data. Typically, model resolution is in the range 200–900 μm, while MRI resolution is 1.3 mm. This means one MRI voxel corresponds to multiple mesh elements.

**Figure 2:**
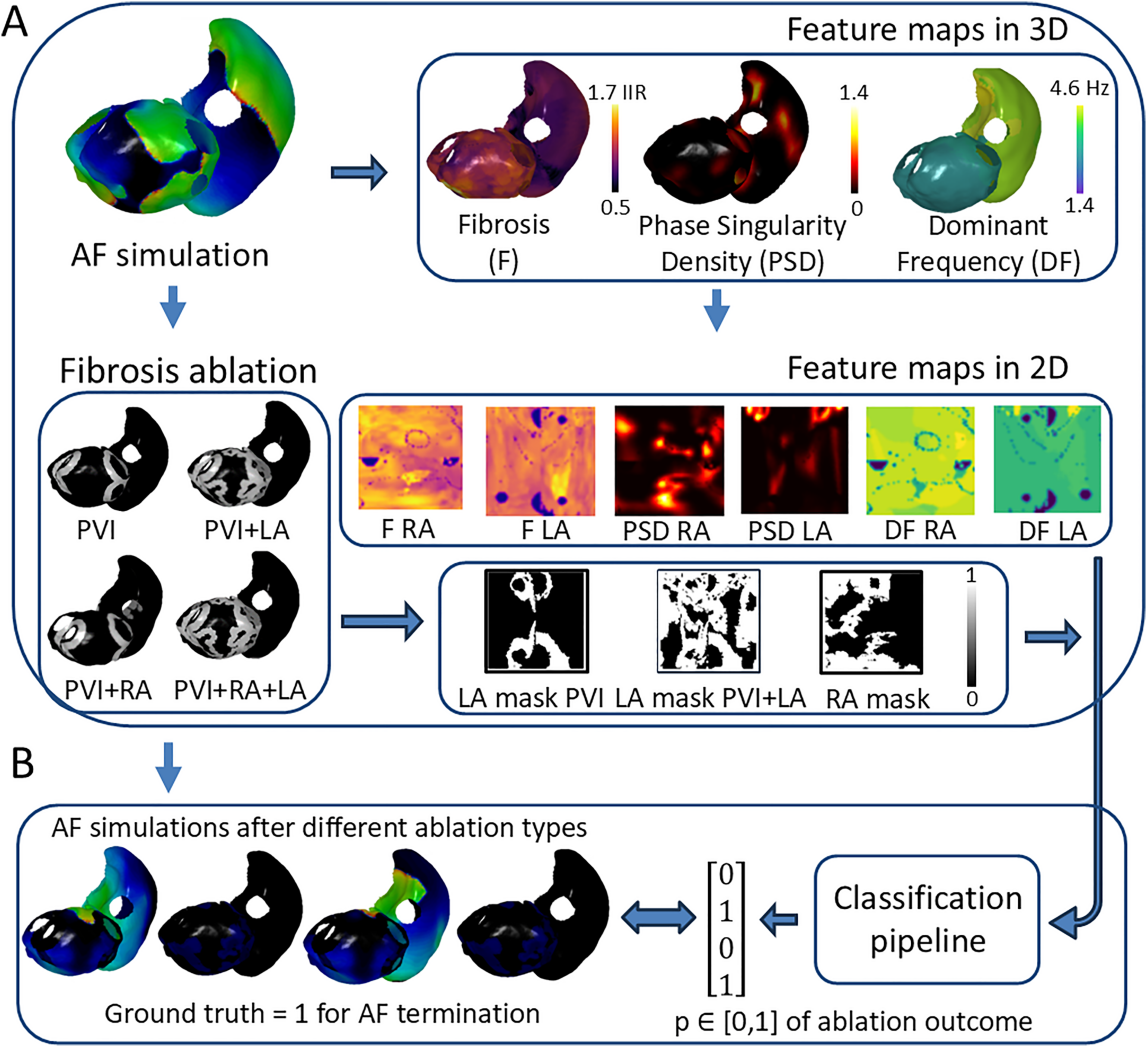
Overview of deep learning pipeline for binary prediction of AF ablation outcome. (**A**) Atrial digital twin based on AF simulation before any ablation provides the feature maps for prediction. (**B**) The proposed pipeline utilizes feature maps and the patient-specific ablation mask to predict the probability of AF termination after ablation, which can be checked by analyzing the AF simulation after ablation. PVI, pulmonary vein isolation; LA, left atrium; RA, right atrium; GT, ground truth label; IIR, image intensity ratio.

To avoid data leakage, we separated the 100 real LGE-MRI fibrosis distributions into three parts, with 80 distributions being assigned for tfhe first training set, 10 for the validation set, and 10 for the testing set. The meshes from the comparison training set were covered with synthetic or noise distributions (described in Section 2.1), resulting in two versions of the comparison training set. All of the aforementioned actions resulted in the creation of an *in silico* dataset of 1,000 virtual patients, which can serve as an initial foundation for biophysical simulations (Figure 2A).

#### 2.2.2 Biophysical simulations of atrial fibrillation episodes

The biophysical simulations were performed by solving the monodomain equation using openCARP software (28) with the Courtemanche ionic model for the cellular action potential (29) to yield transmembrane voltage recordings on each mesh element. Courtemanche et al.’s human atrial cell model was modified in the following way: maximal $I_{Na}$ conductance was multiplied by 2 to ensure physiological action potential upstroke velocities and maximal $I_{K1}$ conductance was multiplied by 0.8 for a closer agreement with clinical restitution data (30). To incorporate the effects of electrical heterogeneity, ionic conductances were modified in each atrial region following Bayer et al. (31). Finally, AF electrical remodeling was incorporated in all atrial regions by reducing the maximal ionic conductances of $I_{to}$, $I_{Kur}$, and $I_{CaL}$ by 50%, 50%, and 70%, respectively, following Courtemanche et al. (32). Tissue conductivities in each atrial region followed the previous studies in which the bilayer model conductivities were chosen to match clinical activation time maps (31, 33).

Fibrotic remodeling was incorporated in the models as regions of conduction slowing (structural remodeling) together with electrophysiological changes (electrophysiological remodeling). This fibrotic remodeling was included depending on the IIR values for both structural and electrophysiological remodeling. For structural remodeling, tissue conductivities in the atrial body were calibrated as follows: IIR<0.9: 0.4 S/m (CV: 0.81 m/s), 0.9<IIR<1.4: 0.31 S/m (CV: 0.74 m/s), 1.4<IIR<1.6: 0.28 S/m (CV: 0.71 m/s), IIR>1.6: 0.19 S/m (CV: 0.58 m/s). For electrophysiological remodeling, ionic properties were modified in fibrotic regions to represent the effects of elevated TGF-ß1 by rescaling maximal ionic conductances as follows: 50% of the regional ionic model value of $g_{K1}$, 60% of $g_{Na}$ and 50% of $g_{CaL}$ (34).

AF was simulated by initially adding four Archimedean spiral waves on the atrial surface for a 15-s duration. The AF simulation was defined as sustained if it had electrical activity for at least 60% of the simulation duration. We modeled four types of ablation strategies, including PVI and PVI together with LA, RA, and bi-atrial fibrosis ablation (Figure 2A). Ablation lesions were applied to the biophysical simulations by setting the corresponding elements of the atrial mesh as non-conducting, with more details in Zolotarev et al. (35). For example, PVI ablations are simulated by adding two non-conducting rings around the left and right pulmonary vein antra.

#### 2.2.3 Feature extraction

**Figure 3:**
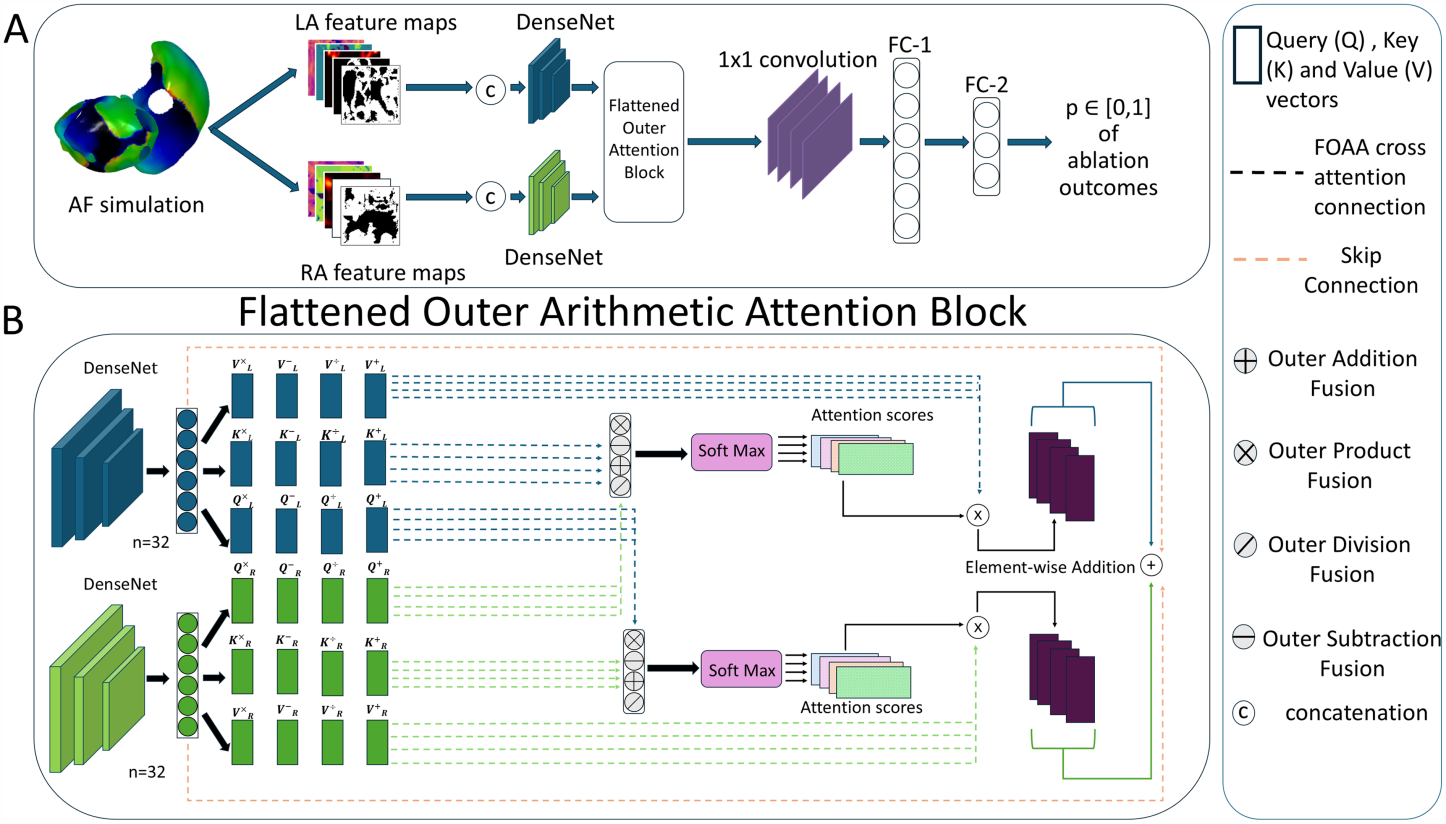
(**A**) Flowchart of deep learning pipeline. LA, left atrium; RA, right atrium; FC, fully connected layer. (**B**) Detailed overview of the flattened outer arithmetic attention (FOAA) block.

The deep learning pipeline was trained based on different input feature maps (Figure 2A). To construct 2D representations of 3D feature maps and feed them into the pipeline, universal atrial coordinates (20) were calculated for each mesh by solving a Laplace equation with boundary conditions. Dominant frequency (DF) and phase singularity density (PSD) maps have been proposed to be relevant attributes to characterize atrial electrical activity (36–38). We have also included the binary ablation masks for each type of ablation. For the left atrium, there were two types of ablation masks: PVI and PVI with left atrial fibrosis ablation. For the right atrium, we included a mask with only right atrial fibrosis ablation. The PVI mask for the right atrium was simulated as an array with all zeros to keep the same number of feature maps in both heads of the proposed DL architecture (Figure 3A).

The DF values were calculated using the frequency of the highest spectral peak of the signal, excluding peaks above 20 Hz. The PSD maps were constructed by counting the number of PS occurrences (31, 39) across the whole duration of the AF simulation at each mesh element and smoothing the values through the mesh. By assigning DF and PSD values from selected mesh elements to corresponding 2D pixels for both LA and RA, we generated four 2D feature maps: DF LA, DF RA, PSD LA, and PSD RA. Finally, we created 2D ablation masks and fibrosis maps (F LA and F RA) based on the same protocol as for the DF and PSD maps (Figure 2A).

The binary outcomes of simulations after different ablation strategies were used to train the DL pipeline and were assigned GT labels, i.e., whether AF terminates or not. We counted the AF episode as terminated if there is no electrical activity at the end of the recording (specifically, the last peak of the action potential is within the range 0%–60% of the whole duration of the recording). To define the final GT, we calculated the last peak’s values of the AF recordings on each mesh element for both the left and right atria and averaged the value across all elements (Figure 2B).

#### 2.2.4 Deep learning pipeline for ablation outcome prediction

We then checked the ability of synthetic fibrosis distributions to imitate the real ones for use in biophysical simulations. We utilized the previously proposed DL pipeline (35) based on Siamese architecture (Figure 3A). Each head of the Siamese architecture consists of a convolutional neural network [DenseNet or ConvNeXt (40)] and utilizes five channel-wise concatenated feature maps (two ablation masks: one for PVI alone and one for PVI with fibrosis region ablation in a specific atrium, PSD, DF, and fibrosis maps, all with the size 96 by 96 pixels) from the left and right atria separately. The outputs of both heads (n=32 each) were fused in a specific manner. The pipeline predicts the outcomes for all ablation strategies at once as four values between 0 and 1 (binary classification for four ablation strategies together rather than for each one separately).

The performance was evaluated by receiver operating characteristic-area under the curve (ROC-AUC) scores on a testing set of 100 virtual patients with fine-tuning of the hyperparameters on the validation set. The training was conducted on an NVIDIA GeForce RTX 3080 video card using binary cross entropy loss and Adam optimizer with a learning rate of 4×10^−7^.

#### 2.2.5 Multi-modal fusion of feature maps

In this study, we explored the use of different multi-modal fusion methods to improve outcome predictions. Previously, we employed simple concatenation and a multi-modal arithmetic block (MOAB) (41). The use of a MOAB allowed for the combination of anatomical features by using outer arithmetic operations. Here, we have introduced a new method: FOAA block (42). Similarly to our previous paper, the FOAA block employs the four outer arithmetic operations but now uses them to replace the scaled-dot product for the calculation of the attention scores (Figure 3B). The latent representations from the two heads were used to derive keys *K*, queries *Q* and values *V* vectors necessary to calculate an attention score as a measure of the similarity between the keys and queries, which was then used to weight the corresponding value vector. The FOAA block also utilises cross-attention (43). This is done through the fusion of the queries from one atria with the queries from the other allowing for the interrelation and combination of every learned feature from the two atria, and therefore improved predictive capacity.

The calculated attention scores from the four outer arithmetic operations were summed and integrated with the single modality features via a skip connection. The output was flattened and then passed through a 1D convolutional layer and two successive fully connected layers (n=48 and 30, respectively). Layer normalization was then performed on the output of the fully connected layers before finally passing through a dropout layer and an activation layer to produce a prediction.

## 3 Results

### 3.1 Artificially generated fibrosis distributions and their statistics

We generated 100 synthetic fibrosis distributions based on a diffusion model trained on 100 real LGE-MRI fibrosis distributions, with examples shown in Figure 1A. The size of both the real and generated images is the same, 96 by 96 pixels. We transferred these distributions to the bi-atrial meshes, with one distribution for the left atrium and one for the right atrium by utilizing *universal atrial coordinates* (20).

**Figure 4:**
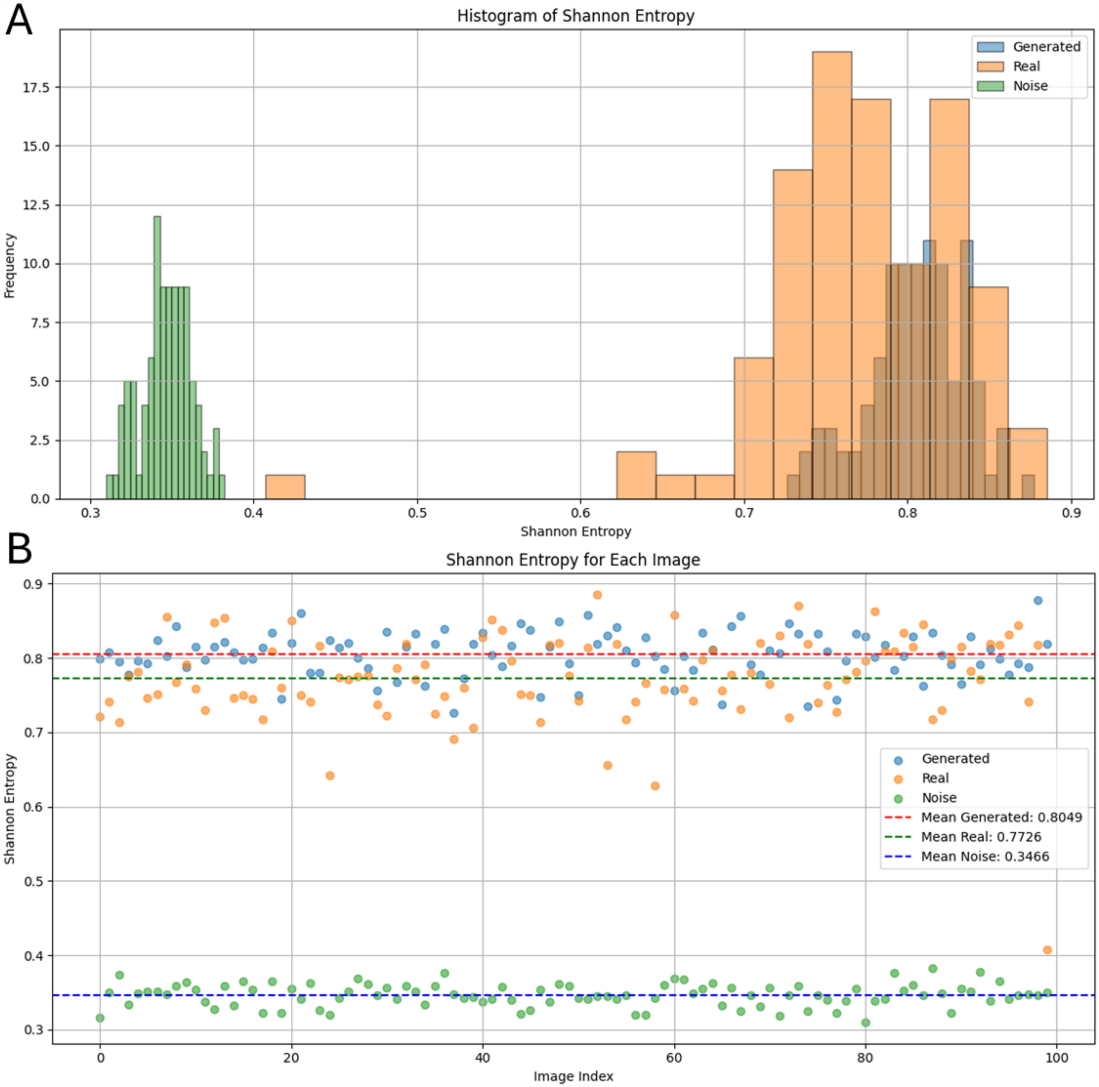
Shannon entropy values of real LGE-MRI, synthetic, and noise fibrosis distributions after applying a Gaussian filter: (**A**) as a histogram; (**B**) individually for each distribution.

Next, we statistically assessed the generated images. First, the synthetic cases were compared with the real LGE-MRI and noise distributions to find possible differences. The mean intensity of the real LGE-MRI distributions was 1.1±0.2 IIR vs. 1.1±0.3 IIR for synthetic distributions and 1.0±0.2 IIR for noise distributions. We next compared them by calculating mean SE after applying a Gaussian filter as a measure of image complexity. The mean SE for the real images was 0.77±0.06 and 0.81±0.03 for synthetic and 0.33±0.02 for the noise images (Figure 4B). Therefore, we can conclude that entropy measurement is an effective tool for separating meaningful images from noise, and that the generated images have a close distribution to the real LGE-MRI ones. However, the standard deviation of entropy for the real LGE-MRI cases was much higher than for the generated and noise fibrotic distributions, which is shown in the histogram in Figure 4A.

### 3.2 Atrial fibrillation biophysical simulations

We then checked the applicability of the generated fibrotic distributions for electrophysiological biophysical simulations on cardiac digital twins. We used the dataset of 1,000 bi-atrial meshes described in Section 2.2.1 to provide anatomical meshes and incorporate the fibrosis distributions on these meshes. We simulated electrophysiological wavefront propagation following AF initialization and after four types of ablation strategies.

We assessed the percentage of sustained AF simulations for all possible datasets and ablation strategies (Table 1). The sustainability of AF was different for different datasets, with the minimum for the testing set (65%) and maximum for the dataset with noise (97%). Overall, the percentage of successful ablations across all tested strategies (which lead to AF termination) varied between 40% for the dataset with synthetic fibrosis and 65% for the training dataset with real LGE-MRI distributions. Analyzing this for the different ablation therapy approaches individually, we found the same pattern for all datasets: the effectiveness of PVI ablation was the lowest, PVI with RA fibrosis ablation was more effective than PVI with LA fibrosis ablation, and PVI with bi-atrial fibrosis ablation remained the most effective (Table 1).

**Table 1:** Values of AF sustainability before and after different ablation strategies.

Percentage of sustained AF recordings	AF	PVI	PVI + LA	PVI + RA	PVI + LA + RA
Training set (real LGE-MRI)	0.86	0.79	0.73	0.65	0.44
Training set (synthetic)	0.66	0.56	0.52	0.34	0.23
Training set (noise)	0.97	0.91	0.83	0.62	0.0025
Validation set	0.71	0.65	0.6	0.41	0.25
Testing set	0.65	0.59	0.54	0.46	0.35

**Figure 6:**
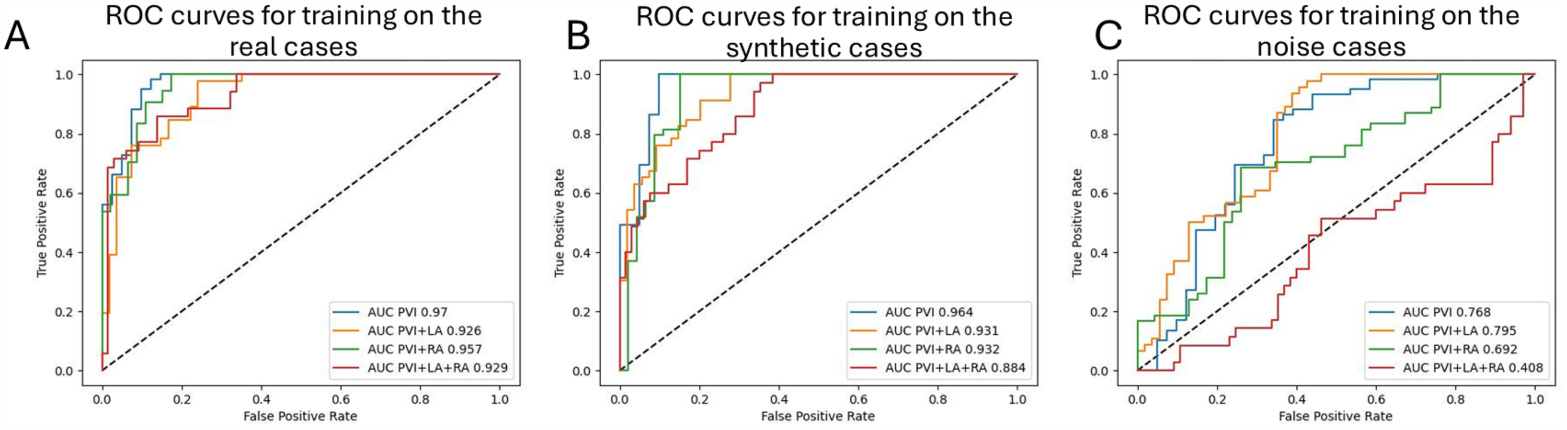
ROC curves with AUCs for the predictions of each simulated ablation strategy (n=4) on the testing set: **(A)** for training on real LGE distributions, **(B)** for training on synthetic distributions, and **(C)** for training on noise distributions.

When analyzing the effect of ablation predictions, it is important to take into account that the mean of AF sustainability before any ablation was different across different datasets (Table 1, first column). Therefore, we also quantified the relative mean of ablation outcome rather than the absolute value of cases with AF termination. For this, we divided the percentage of AF termination after ablation by the percentage of AF sustainability before ablations. We found that the relative percentages of AF sustainability after PVI and PVI with LA fibrosis ablation strategies were very stable across all six datasets (0.91±0.3 for PVI and 0.84±0.03 for PVI+LA, Table 2). The effectiveness of PVI with ablation of fibrosis regions in both atria varies highly, from almost zero for the noise dataset to 53.8% for the testing set. For the training set with noise images, it means that AF terminated in all 400 cases except one case. This unusual distribution can explain the bad performance of this training set for the PVI+LA+RA prediction (Figure 6C).

**Table 2:** Relative effectiveness of AF ablation strategies: percentages of AF sustainability after the specific type of ablation for each dataset and mean percentage across all of them.

Dataset	PVI	PVI + LA	PVI + RA	PVI + LA + RA
Training set (real LGE-MRI)	0.921	0.851	0.763	0.512
Training set (synthetic)	0.855	0.790	0.511	0.344
Training set (noise)	0.943	0.858	0.640	0.003
Validation set	0.915	0.845	0.577	0.352
Testing set	0.908	0.831	0.708	0.538
Mean	0.91	0.84	0.64	0.35
SD	0.03	0.03	0.1	0.21

**Figure 5:**
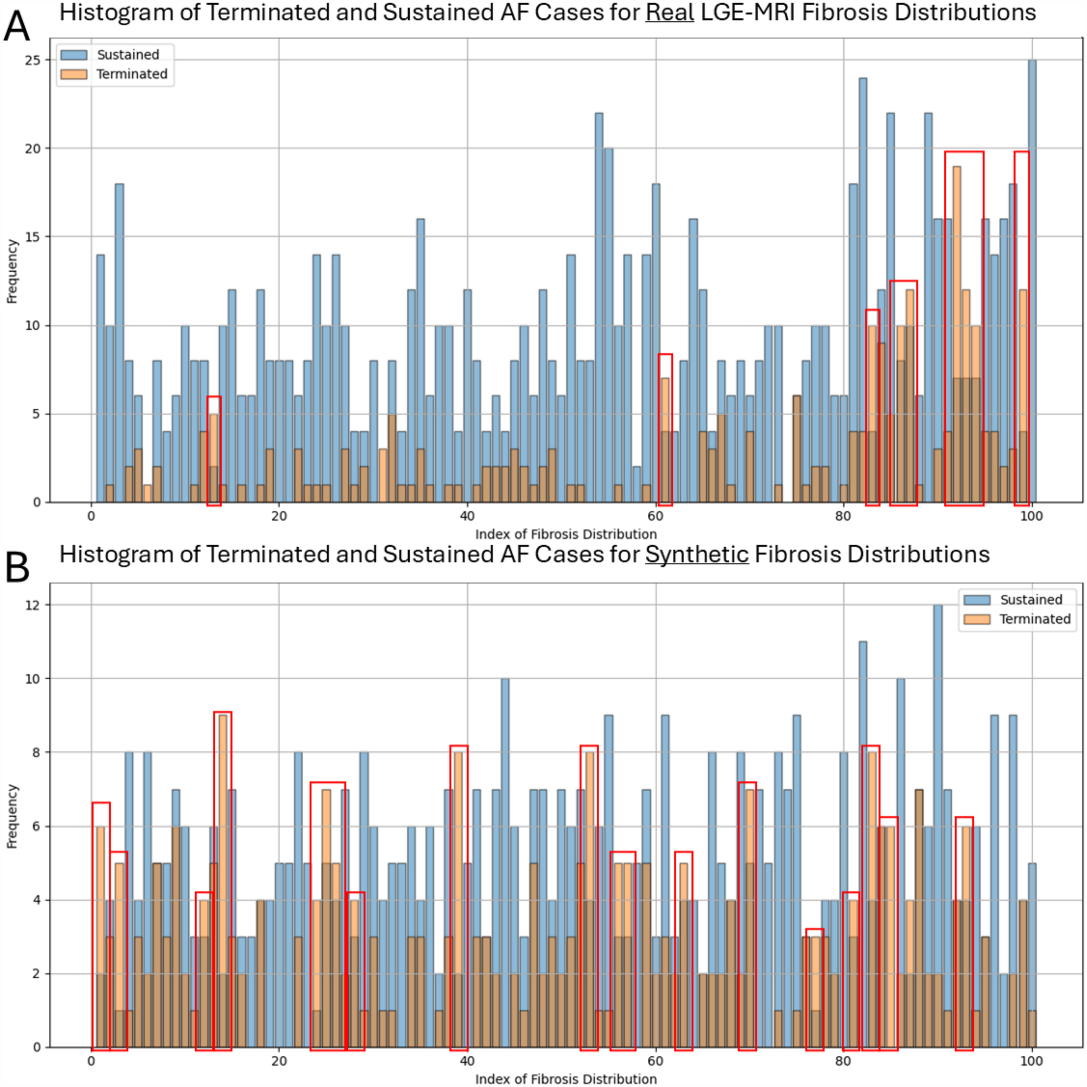
Number of terminated and sustained AF simulations for each fibrosis distribution in two collections of 100 images-real LGE-MRI **(A)** and synthetic **(B)**. Red boxes surrounded the distributions where the number of terminated cases is larger than the number of sustained ones.

Another important aspect is to investigate whether there is any dependency between non-sustained cases and specific fibrosis distributions on these meshes. In other words, whether there are some fibrosis distributions that prevent the AF simulations from being sustained and how they differ from others. We assessed how many times different fibrosis distributions from the collection of 100 LGE-MRI samples, on one side, and from the generated 100 cases, on the other side, are used for the transfer to the bi-atrial meshes. We then separated them into two classes based on whether the AF biophysical simulation with this fibrosis distribution was terminated or sustained even before any ablation (Figure 5). We found that most fibrosis distributions led to sustained cases; however, there were 9 real LGE-MRI and 19 synthetic distributions where the number of terminated cases was larger than the number of sustained ones (these distributions are surrounded by red boxes in Figure 5). The average Shannon entropy for these images was not significantly different from the average SE for the whole set of 100 images (0.76 vs. 0.77 for the real LGE-MRI distributions and 0.81 vs. 0.81 for the synthetic).

### 3.3 Ablation outcome predictions

We have also tested how the prediction of AF ablation outcomes varies depending on the fibrosis distribution. The deep learning pipeline presented in Section 2.2.4 was trained on three different training sets (n=400 meshes) including real LGE-MRI, synthetic, and noise fibrotic distributions. We then fine-tuned the hyperparameters on the validation set of 100 meshes and checked the model performance on the testing set of 100 meshes. The architectures with the highest average ROC-AUC were selected for each training set to show how the performance changes depending on the different fibrosis distributions within the training set. Training on the real LGE-MRI cases secured the highest rank with a ROC-AUC of 0.952 on the testing set (Table 3). The ROC-AUC for training on synthetic distributions was slightly lower (0.943). We used the same DL pipeline based on DenseNet121 and MOAB multi-modal fusion. The training on the noise images achieved a much lower metric (0.682) with the ConvNeXt network and MOAB multi-modal fusion inside.

**Table 3:** ROC-AUC values for prediction of AF ablation outcomes on the testing set.

ROC-AUC	Testing on real LGE-MRI			Testing on synthetic			Testing on noise		
Fusion type	Concat	MOAB	FOAA	Concat	MOAB	FOAA	Concat	MOAB	FOAA
DenseNet	0.911	**0.952**	0.946	0.7	**0.943**	0.928	0.427	0.597	0.666
ConvNeXt	0.932	0.949	0.879	0.52	0.926	0.926	0.51	**0.682**	0.427

On the testing set, the best metrics for each training set is shown in bold.

We present ROC curves and the AUCs for predicting different ablation strategies separately. The curves represent the best-performing models mentioned in the paragraph above. Typically, the PVI outcomes were the easiest to predict, with an AUC of 0.97 for training on the real LGE-MRI distributions and 0.964 for training on the synthetic ones (Figures 6A,B). However, training on noise images showed the best prediction performance for PVI with left atrial fibrosis ablation, with an AUC = 0.795 (Figure 6C). The AUCs for PVI with bi-atrial fibrosis ablations are the lowest for all three scenarios.

We compared alternative configurations to determine the best ROC-AUC for each training set (Table 3). We used two neural network backbones (DenseNet121 and ConvNext) and three approaches for feature fusion (simple concatenation, MOAB, and FOAA). In most cases, DenseNet121 performed better than ConvNext (six items out of nine comparisons, including the highest ROC-AUC). Regarding the fusion technique, MOAB and FOAA had close metrics, with MOAB performing the best when training on real LGE-MRI and synthetic cases, and simple concatenation providing significantly lower metrics.

We also repeated the ablation study for feature maps as in our previous study (38). We utilized the best model (DenseNet121 with MOAB fusion) and tested how changes in input feature maps affect the predictive performance. We found that a full set of feature maps is crucial to achieving the highest prediction performance with the phase singularity density map being the most important (ROC-AUC = 0.732 when used alone, Table 4).

**Table 4:** The effect of input feature maps on the prediction performance. DF, dominant frequency; PSD, phase singularity density.

	Mask	Fibrosis + mask	DF + mask	PSD + mask	Full
ROC-AUC	0.711	0.55	0.668	0.732	**0.946**

On the testing set, the best metrics for each training set is shown in bold.

Finally, we tested how the metrics changed if we trained the model on a mixture of two training sets: the first one consisted of real LGE-MRI and synthetic distributions and the second one consisted of real LGE-MRI and noise distributions. The results for the testing set are shown in Table 5. The highest ROC-AUC for training on the mixture of real LGE-MRI and synthetic cases was 0.946, which was between the highest metrics for training on only real data (0.952) and on only synthetic (0.943). It was obtained using the DenseNet121 network with the simple concatenation of feature maps. Interestingly, the same value was achieved after training the model on a mixture of real LGE-MRI and noise fibrosis distributions (DenseNet with MOAB multi-modal fusion architecture).

**Table 5:** ROC-AUC values for prediction of AF ablation outcomes on the testing set while training the pipeline on the mixture of datasets: combined real LGE with synthetic fibrosis distributions or real LGE with noise fibrosis distributions.

ROC-AUC (test results)	Real LGE-MRI and synthetic			Real LGE-MRI and noise		
Fusion type	Concat	MOAB	FOAA	Concat	MOAB	FOAA
DenseNet	**0.946**	0.896	0.936	0.935	**0.946**	0.927
ConvNeXt	0.931	0.931	0.929	0.931	0.927	0.927

On the testing set, the best metrics for each training set is shown in bold.

## 4 Discussion

We investigated the potential of utilizing synthetic fibrosis distributions to predict the outcomes of AF ablation procedures via a deep learning pipeline trained on feature maps from biophysical simulations of atrial fibrillation. We found that the prediction performance of the deep learning pipeline trained on synthetic fibrosis distributions is comparable to those achieved with real LGE-MRI distributions. The statistical assessment of synthetic and LGE-MRI fibrosis distributions highlights the similarity of these clusters in terms of both mean intensities and Shannon entropies.

We utilized a previously proposed generation architecture (14) that was, to our knowledge, the first implementation of a diffusion model for fibrosis generation. However, the number of cases in our previous study was limited to 100, we did not perform Shannon entropy analysis and we aimed to predict only PVI ablation outcomes by calculating only the dominant frequency feature maps. More recently, we developed a deep learning pipeline with a multi-modal fusion of feature maps from AF simulation before any ablation to predict the outcomes of various types of ablation strategies (35). Our current work combines the best approaches of these two works with key improvements as follows:

• We changed the deep learning pipeline to predict ablation outcomes for all ablation strategies at once (binary classification for four ablation strategies together rather than for each one separately).
• We tested a new convolutional neural network (ConvNeXt) and a new multi-modal fusion (FOAA) block.
• We separated 1,000 bi-atrial meshes and fibrosis distributions into training, validation, and testing sets to avoid data leakage.

We selected the DDPM architecture because it is the simplest diffusion model (U-Net is the standard backbone for diffusion models and we added the attention block to enhance performance) and showed good results in the generation of medical images. However, there are other options to generate images, such as generative adversarial networks (GANs) or VAEs, for example. There are many examples of successful implementations of VAEs for image generation (44). The main disadvantage of VAEs is that the generated images are noisy without clear borders of objects, but this should not be a problem for synthetic fibrotic distributions (even real LGE-MRI distributions have unclear borders of high-intensity clusters, as shown in Figure 1). However, given that diffusion methods have superseded GANs and VAEs in image synthesis (45), we focused on diffusion methods in this article. Future research is needed to compare different generative technologies such as diffusion models, VAEs, and GANs in the creation of artificial fibrosis distributions.

The number of real LGE distributions (n=100) was less than the number of atrial anatomies (n=1,000), therefore some meshes were covered by the same LGE distribution. To avoid data leakage, we separated the 100 fibrosis distributions into three parts, with 80 distributions being assigned for the first training set, 10 for the validation set, and 10 for the testing set. This selection was not stratified and can introduce selection bias, although the ROC-AUC metrics for the validation and testing sets across all experiments were very close (Tables 3 and 7), and, therefore, the effect of selection bias was not significant.

We checked that the generated distributions were statistically close to the real ones by analyzing the mean values of the images and mean Shannon entropies. Moreover, SE was used for the final selection of synthetic cases: we only selected cases with SE >0.66. We also tested another statistical metric (Moran’s I) for the selection, as proposed in Lawson et al. (13). Moran’s I (46) is a measure of spatial auto-correlation, i.e., it informs us of how similar regions in the image are to those around them. However, we observed that this metric was not suitable for our task: the mean Moran’s I was 0.96±0.02, 0.97±0.01, and 0.978±0.001 for the real LGE-MRI, synthetic, and noise fibrosis distributions, respectively.

We calculated how long the training and generating stages of the diffusion model took. Training the DDPM model on 100 images for 500 epochs lasted approximately 3 min while generating 100 synthetic cases took approximately 15 min (10 s per case). To test the approximate times for larger datasets, we generated 1,000 noisy distributions and used them as a training set. The training for the same 500 epochs took 62 min, and generating 1,000 synthetic images using the trained model lasted 150 min. Overall, we can report that training on a 10 times bigger dataset was 20 times longer, while generating cases has the same speed per case (around 10 s). The comparison was conducted using an NVIDIA GeForce RTX 3080 video card.

When analyzing the comparison study on different architectures for the deep learning pipeline, we found that the multi-modal fusion of feature maps always outperformed the simple concatenation. The results of MOAB and FOAA were very close (Table 3). Both provided rich ways to intermingle LA and RA features. However, FOAA’s attention-based strategy did not improve over MOAB, likely because MOAB already captures discriminative features, enabling the model to focus on key information and enhance outcomes. Attention mechanisms, such as those in FOAA, often require a more advanced setup, including multi-headed or gated attention, however, this is left for future work.

We also investigated how consistent the predictive ability of the deep learning pipeline was across repetitive runs via fivefold cross-validation. The training set with real LGE-MRI distributions, which originally consisted of 400 meshes, was separated into four non-overlapping folds of 100 meshes each. The initial validation set consisted of 100 meshes and was counted as the fifth fold. The model was consecutively trained on four folds and then tested on the fifth one, resulting in five ROC-AUC metrics. To simplify the test, we performed the calculation with the best configuration—DenseNet with MOAB fusion. Table 6 presents the achieved results, with a mean ROC-AUC of 0.88±0.03 on the validation folds and 0.94±0.04 on the hold-out testing set. Therefore, the proposed model was stable during the cross-validation experiments.

**Table 6:** ROC-AUC values for the prediction of AF ablation outcomes on fivefold cross-validation, showing the prediction consistency.

ROC-AUC	Fold 1	Fold 2	Fold 3	Fold 4	Fold 5	Mean ± SD
Validation	0.9034	0.8352	0.8783	0.8882	0.8785	0.88±0.03
Test	0.9518	0.9437	0.9426	0.9402	0.9431	0.94±0.04

The deep learning pipeline was trained on four folds and evaluated on the remaining fifth fold (top row) and the hold-out testing set (bottom row).

We have shown that training on synthetic cases leads to significantly better performance rather than training on noise images. However, the training on the mixture of real LGE-MRI and synthetic cases did not provide better results in comparison with training on the mixture of real LGE-MRI and noise fibrosis distributions. In contrast, testing the best models on the validation set showed the expected dynamics (Table 7).

**Table 7:** ROC-AUC values for prediction of AF ablation outcomes on the validation set while training the pipeline on a mixture of datasets.

ROC-AUC	Validation on real LGE-MRI and synthetic			Validation on real LGE-MRI and noise		
Fusion type	Concat	MOAB	FOAA	Concat	MOAB	FOAA
DenseNet	**0.909**	0.891	**0.909**	0.894	0.898	0.889
ConvNeXt	0.883	0.883	0.867	0.863	0.863	0.851

On the testing set, the best metrics for each training set is shown in bold.

Following our approach to predict all outcomes for different ablation strategies at once, our future direction and next goal will be to add more clinically relevant ablation strategies and to find a way to select the optimal ablation strategy. Recently, Sakata et al. (47) showed that not all AF sources should be ablated and some AF drivers lost arrhythmogenic capabilities after other drivers were destroyed. They suggest providing minimum ablation lesions, preventing both AF recurrence and iatrogenic tachycardia.

By following this approach and developing a clinical decision support tool based on deep learning models and synthetic datasets, significant advancements can be achieved in AF treatment. These tools can enhance diagnostic accuracy and personalize treatment plans, potentially leading to improved patient outcomes. Synthetic datasets can help overcome data scarcity and bias, enabling more robust model training and validation. As a result, integrating deep learning-based decision support systems into clinical practice has the potential to optimize AF management and prevent AF recurrence.

### 4.1 Limitations

Our study is not without limitations. First, all findings were based on synthetic SSM anatomies, which may not capture all possible shape variabilities, and further validation on clinical shapes and clinical recordings is needed. Second, AF episodes were initiated by four spiral waves; however, this is not the most common and clinically relevant initiation protocol for AF. Thus, further investigation is needed to compare the AF initiation while performing AF biophysical simulations based on real clinical data. All atrial meshes utilized the same fiber field. The choice of atrial cell model and methodology used to capture fibrotic remodeling will affect AF dynamics and ablation outcome. Future studies are required to investigate these effects across the spectrum of AF dynamics. In total, 1,000 meshes were generated from a limited number of atrial anatomies, and although it was previously shown that the anatomical volumes of this virtual cohort were within the range of values from UK BioBank cohort of over 5,000 individuals (48), the generalizability issue should be tested more carefully on a larger set of clinical meshes. The threshold for creating the ablation mask from fibrosis distributions and the resolution of all feature maps were set up to be 1.22 IIR and 96 pixels, respectively, and changing the values of these hyperparameters may affect the results. The area near the sinoatrial node was not excluded from the RA ablation masks. We aim to predict the acute response after AF ablation; however, AF can be recurrent and appear again months after ablation. Further studies utilizing longitudinal datasets and protocols to evaluate the likelihood of AF initiation and maintenance over time are needed to predict AF recurrence and estimate AF burden. Finally, the sustainability of AF recordings before ablations was not 100%, which helps the deep learning pipeline to predict the ablation outcomes for non-sustained cases.

## 5 Conclusion

To conclude, we explored the potential of using synthetic fibrosis distributions for AF biophysical simulations and predicting outcomes of AF ablation strategies. First, we demonstrated that fibrosis distributions generated by our diffusion model closely resembled actual LGE-MRI distributions, based on metrics such as mean intensities and average Shannon entropy. Second, we confirmed that AF biophysical simulations can be effectively conducted on bi-atrial meshes incorporating these synthetic distributions. Notably, training our deep learning pipeline on these simulations produced performance metrics comparable to those achieved with real LGE-MRI fibrosis distributions. Synthetic fibrosis distributions can help overcome the challenge of limited availability of clinical datasets and enable precise and optimal selection of AF treatment strategy.

## Data Availability

The datasets presented in this study can be found on Zenodo: codes for constructing biatrial bilayer models (https://zenodo.org/records/10139306), CT-derived statistical shape four chamber anatomies (https://zenodo.org/records/4506930) and LGE-MRI fibrosis distributions for left atrial models (https://zenodo.org/records/5801337). OpenCARP solver is an open-source tool for electrophysiology simulations: https://opencarp.org/. Our code for generating fibrosis distribution and running the deep learning pipeline for AF ablation outcome prediction is available on GitHub: https://github.com/pcmlab.
